# Protocol for expressing and purifying recombinant full-length Tau by combining affinity chromatography with preparative HPLC

**DOI:** 10.1016/j.xpro.2026.104617

**Published:** 2026-06-16

**Authors:** Ruiheng Jing, Sayanta Mahapatra, Abhishek Bimali, Morgane Herlory, Maciej A. Walczak

**Affiliations:** 1 University of Colorado, Department of Chemistry, Boulder, CO 80309, USA

**Keywords:** Biophysics, Protein expression and purification, Mass Spectrometry

## Abstract

Deposits of microtubule-associated protein full-length Tau (Tau2N4R) are implicated as a hallmark of Alzheimer’s disease. Its biochemical and structural characterization is key to understanding disease progression, aggregate toxicity, and designing therapeutics. We present a protocol for expressing and purifying Tau2N4R by combining affinity chromatography with preparative high-performance liquid chromatography (HPLC). We describe steps for linking an N-terminal hexahistidine tag and a cleavable SUMO tag to Tau2N4R. This purification ensures an ultrapure Tau2N4R that gives rise to heparin-induced and cofactor-free *in vitro* fibrillation.

## Highlights

• Purification procedure of Tau2N4R from *E. coli* using affinity chromatography and HPLC
• Protocol for efficient His-SUMO tag cleavage from Tau2N4R for downstream experiments
• Step-by-step guide for heparin-induced and cofactor-free fibrillation of Tau2N4R
• Sample preparation and imaging of fibrils by transmission electron microscopy (TEM)

## Abstract (editor highlights)

Publisher’s note: Undertaking any experimental protocol requires adherence to local institutional guidelines for laboratory safety and ethics.

## Graphical abstract

**Figure:**
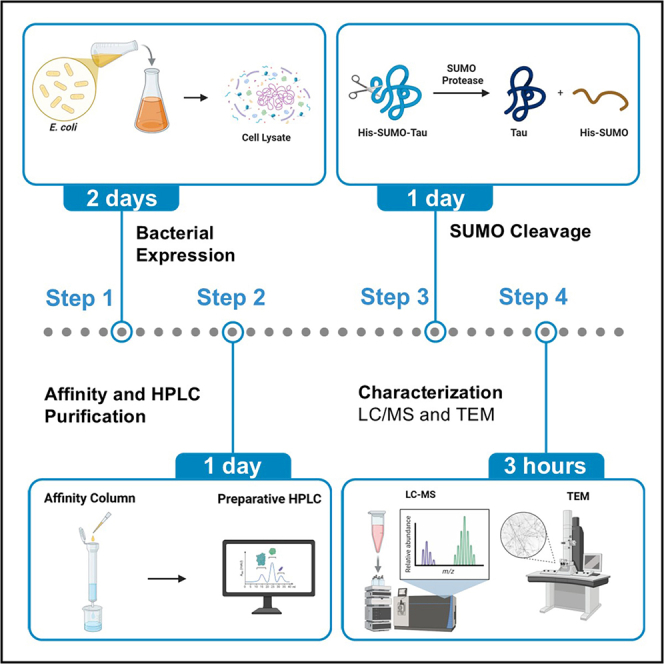

## Before you begin

Prior to initiating the purification, appropriate plasmids, growth media, agar plates, and buffers should be prepared in-house or obtained from commercial suppliers. All reagents must be filtered and/or autoclaved before use to ensure reproducibility and minimize contamination.

### Innovation

This protocol describes a robust and reproducible workflow for purifying recombinant full-length Tau 2N4R isoform (Tau2N4R) bearing an N-terminal hexa-histidine affinity tag linked via a proteolytically cleavable SUMO peptide. Of the six Tau isoforms detected in post-mortem diseased brains, Tau2N4R aggregates are abundant.^1^^,^^2^ While recombinant Tau has been widely used in the field,^3^^,^^4^^,^^5^^,^^6^ many previously reported purification strategies yield material of insufficient purity and heterogeneity that limits its suitability for high-precision biophysical and structural studies. In particular, prior approaches often produce Tau preparations that are not homogeneous by high-resolution mass spectrometry (HRMS), reflecting co-purified truncations, post-expression modifications, or residual fusion-tag–derived species. Chemical methods for the *de novo* synthesis of Tau2N4R and Tau with post-translational modifications are known,^7^^,^^8^ yield material of high purity and devoid of any toxins, but these methods are time consuming and require specialized equipment.

Here, we detail a step-by-step protocol for the expression and purification of His-SUMO-Tau2N4R from *E. coli*, followed by efficient SUMO protease cleavage and sequential chromatographic purification steps designed to remove incompletely cleaved species, degradation products, and trace contaminants.^9^ The resulting Tau2N4R is obtained at high purity and exhibits a single, well-defined molecular species by HRMS, enabling its direct use in biophysical assays and *in vitro* fibrillation studies where molecular homogeneity is essential.

## Key resources table

**Table:** 

REAGENT or RESOURCE	SOURCE	IDENTIFIER
**Bacterial and viral strains**		

*Escherichia coli* BL21 (DE3)	New England Biolabs	Cat # C2527H

**Chemicals, peptides, and recombinant proteins**		

HEPES Free Acid	RPI	CAS: # 7365-45-9
Sodium Chloride	RPI	CAS: # 7647-14-5
Imidazole	RPI	CAS: # 288-32-4
Sodium Phosphate Monobasic	Sigma-Aldrich	CAS: # 7558-80-7
Sodium Phosphate Dibasic	Sigma-Aldrich	CAS: # 7558-79-4
Heparin Sodium Salt (18 kDa)	Sigma-Aldrich	CAS: # 9041-08-1
Formic Acid (FA)	Sigma-Aldrich	CAS: # 64-18-6
Acetic Acid	MilliporeSigma	CAS: # 64-19-7
Trifluoroacetic Acid (TFA)	ChemImpex	CAS: # 76-05-1
Methanol	Thermo Fisher Scientific	CAS: # 67-56-1
Acetonitrile (MeCN)	VWR	Cat # JT9017-3
Dithiothreitol (DTT)	RPI	CAS: # 3483-12-3
2-Mercaptoethanol	Bio-Rad	Cat # 1610710
Uranyl Acetate	Electron Microscopy Sciences	CAS: # 541-09-3
Milli-Q water	MilliporeSigma	Cat # ZIQ7010T0
Pierce Protease Inhibitor Mini Tablets, EDTA-free	Thermo Fisher Scientific	Cat # A32955
Protein ladder for Ni-IMAC	Thermo Fisher Scientific	Cat # 26619
Sodium Hydroxide Pellets	Fisher Chemical	Cat # S318-3
SOC Outgrowth Media	New England Biolabs	Cat # B9020S
LB Agar (Lennox Agar)	RPI	CAS: # 91079-40-2
Luria Broth (Miller’s LB Broth)	RPI	CAS: # 8013-01-2
Kanamycin Monosulfate	RPI	CAS: # 25389-94-0
Isopropyl β-D-thiogalactopyranoside	RPI	CAS: # 367-93-1
Lysozyme from Chicken Egg White, Salt Free	RPI	CAS: # 12650-88-3
Ni-NTA Agarose Resin	Qiagen	Cat # 30210
1 M Magnesium Chloride	Invitrogen	Cat # AM9530G
10% Mini-PROTEAN TGX PreCast Gels	Bio-Rad	Cat # 4561033
10 × Tris/Glycine/SDS Solution	Bio-Rad	Cat # 1610772EDU
Precision Plus Protein Standards	Bio-Rad	Cat # 1610374
Coomassie Brilliant Blue R-250	Bio-Rad	Cat # 1610400
4 × Laemmli Sample Buffer	Bio-Rad	Cat # 1610747
Water, sterile-filtered, BioReagent	Sigma-Aldrich	Cat # W3500-500 mL

**Recombinant DNA**		

pET30a(+)-His-SUMO-Tau2N4R	GenScript	N/A
pET-28b(+)-pFGET19_Ulp1 (His-Ulp1)	Addgene	Cat # 64697

**Other**		

Innova 42R Refrigerated Incubator Shaker	New Brunswick	Cat # 05400162
L-Shaped Cell Spreaders Sterile	Fisher Scientific	Cat # 14-665-231
ACCQ HP-150 preparative HPLC	Teledyne	N/A
1260 Infinity II/G6125B LC/MSD	Agilent	N/A
10 mm UV Cuvette	Sigma-Aldrich	Cat # Z628026
Nalgene Oak Ridge High-Speed PPCO Centrifuge Tubes	Nalgene	Cat # 3110-0500
SpectraMax iD5 Plate Reader	Molecular Devices	N/A
FC5916 120 V Centrifuge	Ohaus	Cat # 30553037
Eppendorf Centrifuge 5804R	Eppendorf	Cat # 05-400-93
Rocking Shaker	Ohaus	Cat # 30391959
Precision Shaking Water Bath	Thermo Scientific	Cat # TSSWB15
Nunc Polypropylene Conical Centrifuge Tubes 15 mL	Thermo Scientific	Cat # 339650
Nunc Polypropylene Conical Centrifuge Tubes 50 mL	Thermo Scientific	Cat # 339653
Petri Dish	VWR	Cat # NC1936598
MiniSpin Plus Mini Centrifuge	Eppendorf	Cat # 5453000015
Ward’s Mini Centrifuge	Ward’s Science	Cat # 470356-704
Digital Heatblock	VWR	Cat # 460-0350
1.7 mL MCT Graduated Clear Tubes	VWR	Cat # 490004-436
0.65 mL MCT Graduated Clear Tubes	VWR	Cat # 490004-456
Parafilm Laboratory Film	Bemis	Cat # HS234526B
QSonica Q500 Probe Sonicator	QSonica	Cat # Q500-110
Corning Bottle-Top Vacuum Filters	Corning	Cat # CLS430513
Amicon 0.5 mL Ultra Centrifugal Filter 3 kDa	MilliporeSigma	Cat # UFC5003
Formvar/Carbon 200 Mesh, Copper	Electron Microscopy Sciences	Cat # FCF200-Cu-50
Mini PROTEAN Tetra Cell	Bio-Rad	Cat # 1658004
OSP-250 L Power Supply	Owl Separation Systems	Cat # 250900SP
800 μM Silica Beads	OPS Diagnostics	Cat # NC1468556
90 mm Qualitative Filter Paper Grade 4	Whatman	Cat # WHA1004090
Econo-Pac Chromatography Columns	Bio-Rad	Cat # 7321010
Corning 384 Well Microplate	Corning	Cat # CLS3985
EMITECH K400× Carbon Coater	EMITECH	N/A
ChemiDoc Imaging System	Bio-Rad	Cat # 12003153
VANTAstar Flexible Multi-mode Microplate Reader	BMG Labtech	N/A
Waters C4 column (19 mm × 250 mm, 300 Å, 5 μm)	Waters	Cat # 186007314
Agilent EC-C18 Poroshell column (4.6 × 100 mm, 100 Å, 4 μm)	Agilent	Cat # 693970-902
15 mL plastic chromatography gravity column	Bio-Rad	Cat # 7321010EDU
0.2 μm SFCA filter	Nalgene	Cat # 291-4520
0.22 μm Syringe Filters Nylon (25 mm)	Adamas-beta	Cat # 02036320-TYLQ-0003
Borosilicate Glass tube (18 × 150 mm)	Fisher Scientific	Cat # 14-961-32

## Materials and equipment

**Table:** His-Ulp1 Lysis Buffer∗

Reagent	Final concentration
Sodium Phosphate∗ (pH 7.4)	50 mM
NaCl	300 mM
Imidazole	10 mM

[Store at 4°C for up to 1 month]

**Table:** His-SUMO-Tau2N4R Lysis Buffer

Reagent	Final concentration
HEPES (pH 7.4)	25 mM
NaCl	300 mM
Imidazole	20 mM

[Store at 4°C for up to 1 month].

**Table:** Cofactor-free Tau2N4R Aggregation Buffer∗

Reagent	Final concentration
Sodium Phosphate∗ (pH 7.4)	10 mM
DTT	10 mM
MgCl_2_	20 mM

[Store at 4°C for up to 1 month; DTT is added immediately to stored buffer prior to use].

**Table:** Heparin-Induced Tau2N4R Aggregation Buffer

Reagent	Final concentration
Heparin (18 kDa)	5 μM
Thioflavin T (ThT)	20 μM
NaCl	50 mM
DTT	5 mM
HEPES (pH 7.4)	25 mM

[Store at 4°C for up to 1 month; Heparin and DTT are added immediately to stored buffer prior to use].

∗Sodium Phosphate Buffers made using Monosodium and Disodium Phosphate using Michigan State University Buffer Calculator.

***Note:*** Filter all the buffers using bottle-top vacuum filters (Corning) prior to use.

## Step-by-step method details

### Transformation of pET30a(+)-His-SUMO-Tau2N4R into BL21(DE3) cells using the heat shock method

**Timing: 2 days**

This section describes the protocol for transformation of a plasmid encoding His-SUMO-Tau2N4R into chemically competent *E. coli* cells.

**Figure 1:**
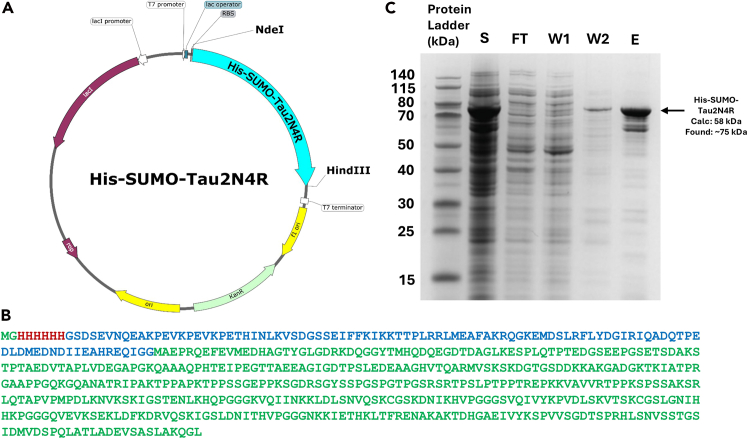
Design of the plasmid and affinity purification of His-SUMO-Tau2N4R (A) Detailed plasmid map of pET30a(+)-His-SUMO-Tau2N4R showing the vector [pET30a(+)] and insertion (His-SUMO-Tau2N4R) in blue. (B) Amino acid sequence of His-SUMO-Tau2N4R showing the hexa-histidine tag (in red) and SUMO tag (in blue). (C) SDS-PAGE of Ni-IMAC of His-SUMO-Tau2N4R. Lanes for supernatant, flow-through, wash 1, wash 2, and elution are marked as S, FT, W1, W2, and E, respectively. Tau protein shows a higher molecular weight than calculated in the gel.^10^

***Note:*** The expression construct was designed in-house, then codon optimized and synthesized by a commercial vendor (GenScript). The plasmid structure and primary amino acid sequence for Tau2N4R (UniProt accession number: P10636-8) are shown in Figures 1A and 1B.

1. Mix pET30a(+)-His-SUMO-Tau2N4R plasmid (1 pg–100 ng) with 25 μL of freshly thawed (on ice) chemically competent BL21(DE3) cells (New England Biolabs) and incubate on ice for 30 min.

***Note:*** Mix by gently tapping the bottom of the tube and avoid pipetting.

2. Transfer the tube to a 42°C water bath (Thermo Scientific) for 10 s and then quickly cool it by putting on ice for 5 min.
3. Add 450 μL of SOC outgrowth medium to the tube and shake at 220 rpm, 37°C (New Brunswick Innova 42R) for 1 h.
4. Spread 100 μL of the culture on an LB agar plate containing 50 μg/mL kanamycin and spread it evenly on the plate using a L-shaped cell spreader (Fisher Scientific).
5. Incubate for 16–24 h at 37°C in the incubator.

***Note:*** If the plasmid transformation efficiency is low, see troubleshooting, problem 1.

### Grow the transformed cells and induce His-SUMO-Tau2N4R expression

**Timing: 2 days**

This section describes the growth of the transformed cells and the over-expression of His-SUMO-Tau2N4R protein.

***Note:*** The protein overexpression was induced with isopropyl β-D-1-thiogalactopyranoside (IPTG).

6. Pick a single colony, set up 40 mL primary culture in LB supplemented with 50 μg/mL kanamycin and grow for 16–18 h at 37°C with shaking at 220 rpm (New Brunswick Innova 42R).
7. For secondary culture, transfer 4 × 10 mL primary culture to 4 × 1 L LB (with 50 μg/mL kanamycin) such that 10 mL of primary culture/liter of secondary culture.
  a. Grow the cells at 37°C with shaking at 220 rpm (New Brunswick Innova 42R).
  b. Test the OD_600_ until it reaches 0.4–0.6.
8. Introduce 1 mM IPTG to induce protein expression for 3 h at 37°C and 220 rpm.
9. Harvest cells by centrifugation (4350 × *g*, 30 min) (Ohaus FC5916 Centrifuge).

***Note:*** The lysate can be stored at −80°C for one month after harvesting.

***Note:*** The cell pellet from each secondary culture can be stored and processed together.

### His-SUMO-Tau2N4R purification by nickel-immobilized metal affinity chromatography

**Timing: 1 day**

This section describes a detailed protocol for the harvest and lyse of *E. coli* cells and the isolation of His-SUMO-Tau2N4R protein from host-cell proteins using Ni-IMAC.

10. Resuspend harvested cells (∼10 g) in 20 mL of His-SUMO-Tau2N4R lysis buffer containing protease inhibitors (Pierce Protease Inhibitor Tablets, Thermo Scientific).
11. Lyse cells by incubating the cell suspension with lysozyme (1.5 mg/mL, RPI) for 2 h.
  a. Sonicate the cell suspension (55% amplitude, 30 s pulse on, 30 s pulse off for a total time of 12 min, QSonica Q500 Probe Sonicator) on ice to avoid heating.
12. Separate the cell debris by centrifugation at 4°C, 15,000 × *g* for 30 min (Eppendorf Centrifuge 5804R).
  a. Collect the supernatant that contains the protein.
  b. Save 15 μL of the supernatant for SDS-PAGE analysis.
13. Equilibrate 4 mL of Ni-NTA agarose resin (Qiagen) with the His-SUMO-Tau2N4R lysis buffer in 50 mL tubes.
14. Mix the resin with the supernatant, incubate this mixture with gentle agitation in a nutating mixer for 2 h at 4°C.
15. Transfer the mixture to a 15 mL plastic chromatography gravity column (Bio-Rad).
  a. Collect the flow-through from the column (FT).
  b. Save 15 μL of the flow-through for SDS-PAGE analysis.
16. Wash the resin with 50 mL of His-SUMO-Tau2N4R lysis buffer with 20 mM imidazole at 4°C, collected as wash 1 (W1).

***Note:*** Using less wash buffer may not effectively remove contaminants.

17. Wash the resin again with 50 mL His-SUMO-Tau2N4R lysis buffer supplemented with 30 mM imidazole at 4°C, collected as wash 2 (W2).
18. Elute bound His-SUMO-Tau2N4R using 50 mL of His-SUMO-Tau2N4R elution buffer (His-SUMO-Tau2N4R lysis buffer supplemented with 280 mM imidazole), collected as elution (E).

***Note:*** Elute the resin with less buffer may not collect all the proteins.

**Pause point:** The elution fraction can be stored at 4°C for up to 24 h.

19. Add 5 μL of 4 × SDS-loading dye mixed with 2-mercaptoethanol to each 15 μL sample.
  a. Boil them at 90°C for 4 min.
  b. Use the Precision Plus Protein Dual Color Standards (From Biorad #1610374).
  c. Run an SDS-PAGE (225 Volts, ∼25 min, Owl Separation Systems) to check the protein purity.
20. Stain the gel with 0.5% Coomassie Blue R-250 in 50% methanol and 10% acetic acid for 30 min.
  a. Destain the gel in water/destaining solution (50% methanol, 10% acetic acid in water) until the protein bands appear.
  b. Image the gel using a ChemiDoc Gel Imager (Bio-Rad), Figure 1C.

### His-SUMO-Tau2N4R purification by high-performance liquid chromatography

**Timing: 3–5 h**

This section described the purification protocol for His-SUMO-Tau2N4R protein using HPLC.

21. Purify 50 mL of His-SUMO-Tau2N4R elution from the Ni-IMAC column on a Teledyne HP-150 preparative HPLC.

***Note:*** Filter the elution through a 0.2 μm SFCA filter (Nalgene, Cat. No. 291-4520) under vacuum before loading.

22. Load 50 mL of filtered elution on Waters C4 column (19 mm × 250 mm, 300 Å, 5 μm, 25°C, 17.1 mL/min) using the gradient in Table 1.

**Table 1:** HPLC gradient for His-SUMO-Tau2N4R purification

Time	H_2_O/0.1% TFA	MeCN/0.1% TFA
0 min	90%	10%
7 min	90%	10%
9.7 min	80%	20%
49.7 min	40%	60%
52.4 min	0%	100%
57.4 min	0%	100%

***Note:*** Load the column using the multiple-injection method on the HP-150. Load 5 injections of 9 mL, then the final injection of 5 mL. Flush the column with 10% MeCN/H_2_O/0.1% TFA for 7 min after each injection to remove sodium phosphate, NaCl, and imidazole.

23. Collect the protein which eluted at 35%–40% MeCN/H_2_O/0.1% TFA from the C4 column.
  a. Take 10 μL from each fraction for Liquid Chromatography-Mass Spectroscopy (LC-MS) analysis.

***Note:*** Collect 3–6 fractions based on the absorbance at 214 nm on HP-150.

***Note:*** Collect 15 mL in each fraction for the best separation.

24. Analyze the fractions using Agilent LC/MSD on Agilent EC-C18 Poroshell column (4.6 × 100 mm, 4 μm, 100 Å, 40°C, 1 mL/min) using the gradient in Table 2.

**Table 2:** LC gradient for fractions from HPLC of His-SUMO-Tau2N4R

Time	H_2_O/0.1% FA	MeCN/0.1% FA
0 min	95%	5%
5 min	5%	95%
7 min	5%	95%
7.1 min	95%	5%
9 min	95%	5%
25. Combine the fractions containing the product in 50 mL conical tubes.
  a. Freeze the tubes with liquid nitrogen and lyophilize the solution to provide 15 mg lyophilized His-SUMO-Tau2N4R as a white solid.

***Note:*** Any LC/MS instrument capable of performing analytical HPLC is sufficient to analyze the samples.

**Pause point:** The lyophilized protein can be stored at −20°C for up to 7 days.

### Transformation of pET28b(+)-His-Ulp1 into BL21(DE3) cells using the heat shock method

**Timing: 2 days**

This section describes the protocol for transformation of a plasmid encoding His-Ulp1 into chemically competent *E. coli* cells.

***Note:*** Ulp1 is a SUMO protease widely used to proteolytically cleave affinity tags from recombinant proteins. The purified Ulp1 is used to cleave the His-SUMO moiety from Tau2N4R in the upcoming steps. The plasmid is obtained from Addgene (Plasmid #64697) as pET28b(+)-His-Ulp1.

26. Mix pET28b(+)-His-Ulp1 plasmid (1 pg–100 ng) with 25 μL of freshly thawed (on ice) chemically competent BL21(DE3) cells (New England Biolabs) and keep on ice for 30 min.

***Note:*** Mix gently by tapping the bottom of the tube and avoid pipetting.

27. Briefly heat-shock the cells in a 42°C water bath (Thermo Scientific) for 10 s and then quickly cool them down by putting them on ice for 5 min.
28. Add 450 μL of SOC outgrowth medium to the tube and shake at 220 rpm, 37°C (New Brunswick Innova 42R) for 1 h.
29. Spread 100 μL of the above culture uniformly on the plate using a L-shaped cell spreader (Fisher Scientific) on an LB agar plate containing 50 μg/mL kanamycin.
30. Incubate for 16–24 h at 37°C.

***Note:*** If the plasmid transformation efficiency is low, see troubleshooting, problem 1.

### Grow the transformed cells and induce His-Ulp1 expression

**Timing: 1 day**

This section describes the growth of the transformed cells and the over-expression of His-Ulp1 protein.

31. Pick a single colony from freshly transformed plates and add it to 20 mL of primary culture in LB supplemented with 50 μg/mL kanamycin and grow for 16–18 h at 37°C with shaking at 220 rpm (New Brunswick Innova 42R).
32. To set up secondary culture, transfer 2 × 10 mL of primary culture to 2 × 1 L LB (with 50 μg/mL kanamycin) such that 10 mL of primary culture/liter of secondary culture.
  a. Grow the cells at 37°C with shaking at 220 rpm (New Brunswick Innova 42R).
  b. Test the OD_600_ until it reaches 0.4–0.6.
33. Add 500 μM IPTG to induce protein expression for 3 h at 37°C and 220 rpm.
34. Harvest cells by centrifugation (4350 × *g*, 30 min) (Ohaus FC5916 Centrifuge).

### His-Ulp1 purification by Ni-IMAC

**Timing: 1 day**

This section describes a detailed protocol for the harvest and lyse of *E. coli* cells and the isolation of His-Ulp1 protein from host-cell proteins using Ni-IMAC.

**Figure 2:**
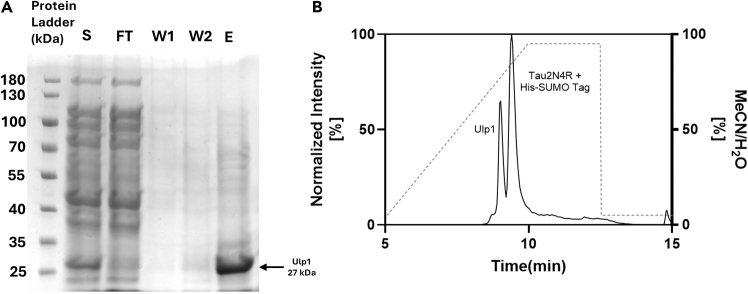
Purification of SUMO protease and cleavage of His-SUMO tag from His-SUMO-Tau2N4R (A) SDS-PAGE of Ni-IMAC of SUMO protease Ulp1. Lanes for supernatant, flow-through, wash 1, wash 2, and elution are marked as S, FT, W1, W2, and E, respectively. (B) Normalized total ion chromatography of SUMO tag cleavage reaction. Dash line represents the LC gradient.

35. Homogenize harvested cells (∼4 g) in 20 mL of His-Ulp1 lysis buffer having protease inhibitors (Pierce Protease Inhibitor Tablets, Thermo Scientific).
36. Lyse cells by incubating the cell homogenate with lysozyme (1.5 mg/mL) (RPI) for 2 h.
  a. Sonicate the cell suspension (55% amplitude, 30 s pulse on, 30 s pulse off for a total on time of 12 min, QSonica Q500 Probe Sonicator) on ice to avoid heating.
37. Isolate the cell debris by centrifugation at 4°C, 15,000 × *g* for 30 min (Eppendorf Centrifuge 5804R), and collect the supernatant that contains the protein (S).
  a. Save 15 μL of the supernatant for SDS-PAGE analysis.
38. Equilibrate 3 mL of Ni-NTA agarose resin (Qiagen) with the His-Ulp1 lysis buffer in 50 mL tubes.
39. Mix the resin with the supernatant, incubate this mixture with gentle agitation in a nutating mixer for 2 h at 4°C.
40. Transfer the mixture to a 15 mL plastic chromatography gravity column (Bio-Rad) and collect the flow-through from the column (FT).
  a. Save 15 μL of supernatant and flow-through for SDS-PAGE analysis.
41. Wash the resin with 40 mL of His-Ulp1 lysis buffer with 10 mM imidazole at 4°C, collected as wash 1 (W1).
42. Wash the resin with 10 mL of His-Ulp1 lysis buffer supplemented with 40 mM imidazole, collected as wash 2 (W2).
43. Elute bound His-Ulp1 using 10 mL of His-Ulp1 elution buffer (His-Ulp1 lysis buffer supplemented with 280 mM imidazole), collected as elution (E).
44. Add 5 μL of 4 × SDS-loading dye mixed with 2-mercaptoethanol (Bio-Rad) to each 15 μL sample.
  a. Boil them at 90°C for 4 min,
  b. Use the molecular weight marker in the gel.
  c. Run an SDS-PAGE (225 V, ∼25 min, Owl Separation Systems) to check the protein purity.
45. Stain the gel with 0.5% Coomassie Blue R-250 in 50% methanol and 10% acetic acid for 30 min.
  a. Destain the gel with water/destaining solution (50% methanol and 10% acetic acid in water) until the protein bands appear.
  b. Image the gel using a ChemiDoc Gel Imager (Bio-Rad), Figure 2A.

**Figure 3:**
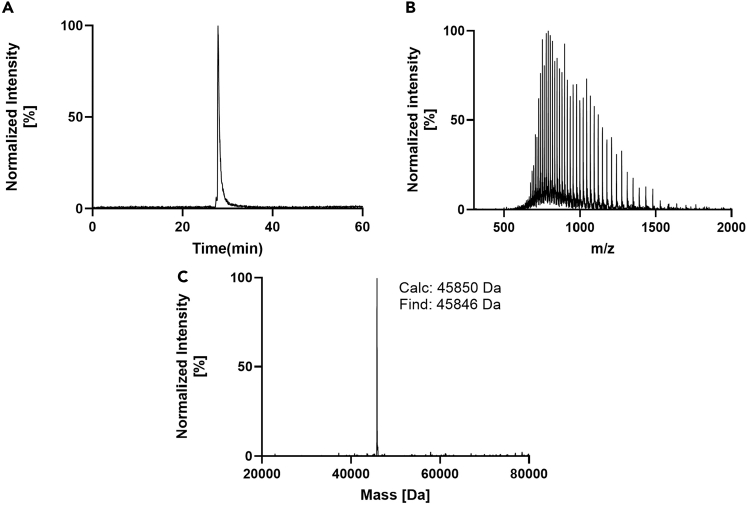
Characterization of Tau2N4R (A) Normalized total ion chromatography of HPLC-purified Tau2N4R protein. (B) Normalized ESI(+) mass spectrometry (300–2000 m/z) of HPLC-purified Tau2N4R protein. (C) Normalized deconvoluted mass of HPLC-purified Tau2N4R protein.
46. Use liquid nitrogen to flash freeze the protein in elution buffer, store the protein in−80°C.

### His-SUMO tag cleavage of His-SUMO-Tau2N4R

**Timing: 1 day**

This section describes a detailed protocol for His-SUMO tag cleavage of His-SUMO-Tau2N4R using His-Ulp1 protein.

47. Dissolve 15 mg of lyophilized His-SUMO-Tau2N4R in 10 mL of 10 mM sodium phosphate buffer, pH 7.4.
  a. Perform a test reaction to test the activity of the Ulp-1 protease.

**CRITICAL:** The activity of Ulp1 protease changes from batch to batch. A test reaction is needed for each batch of Ulp1 protease.

48. Add 1% Ulp1 protease (molar basis) to 100 μL of protein solution.
  a. Incubate the reaction in a 0.65 mL Eppendorf tube at 23°C for 16 h.
  b. Take 10 μL of the solution for LC-MS analysis.
49. Monitor reaction progress using Agilent LC/MSD on Agilent EC-C18 Poroshell column (4.6 × 100 mm, 4 μm, 100 Å, 40°C, 1 mL/min) using the gradient in Table 3.

**Table 3:** LC gradient for His-SUMO tag cleavage

Time	H_2_O/0.1% TFA	MeCN/0.1% TFA
0 min	95%	5%
5 min	95%	5%
10 min	5%	95%
12 min	5%	95%
12.1 min	95%	5%
15 min	95%	5%

**CRITICAL:** If the reaction is not completed, more Ulp1 protease can be added to the reaction, and the reaction progress needs to be tested again after 16 h. The same molar ratio of protease and protein should be applied to the large scale of the reaction (see troubleshooting, problem 2).

50. Add 1% Ulp1 protease (molar basis) to the 10 mL protein solution and incubate the reaction in a 50 mL conical tube at 23°C for 16 h.
  a. Take 10 μL of the solution for LC-MS analysis.

**CRITICAL:** If the reaction turbid due to a high protein concentration, add 10 mL of 10 mM sodium phosphate buffer to perform the reaction as a clear solution (see troubleshooting, problem 3).

51. Monitor reaction progress using Agilent LC/MSD on Agilent EC-C18 Poroshell column (4.6 × 100 mm, 4 μm, 100 Å, 40°C, 1 mL/min) using the gradient in Table 3.

***Note:*** The completion of the reaction needs to be tested by LC-MS, Figure 2B.

### HPLC purification of Tau2N4R

**Timing: 3–5 h**

This section describes a detailed protocol for purification of Tau2N4R protein using HPLC.

52. Purify 10 mL of His-SUMO tag cleavage reaction on Teledyne HP-150 preparative HPLC.
  a. Filter the reaction through 0.2 μm SFCA filter (Nalgene, Cat. No. 291-4520) under vacuum before loading on HPLC.
53. Load the sample on Waters C4 column (19 mm × 250 mm, 300 Å, 5 μm, 25°C, 17.1 mL/min) using the gradient in Table 4.

**Table 4:** LC gradient for fractions from HPLC of Tau2N4R

Time	H_2_O/0.1% FA	MeCN/0.1% FA
0 min	90%	10%
7 min	90%	10%
9.7 min	80%	20%
49.7min	40%	60%
52.4 min	0%	100%
57.4 min	0%	100%
54. Collect the protein which elutes at 35%–40% MeCN/H_2_O/0.1% TFA from the C4 column.
  a. Take 10 μL aliquots from each fraction for LC-MS analysis.

***Note:*** Collect 3–6 fractions based on the absorbance at 214 nm on HP-150.

***Note:*** Collect 15 mL fractions for the best separation.

55. Analyze the fractions using Agilent LC/MSD on Agilent EC-C18 Poroshell column (4.6 × 100 mm, 4 μm, 100 Å, 40°C, 1 mL/min), using the gradient in Table 2.
56. Combine the fractions containing the product in 50 mL conical tubes.
  a. Freeze the tubes with liquid nitrogen and lyophilize to provide 10 mg of lyophilized Tau2N4R as a white solid.

***Note:*** The reproducibility of the purification step is proved in Extended Figure S1.

**Pause point:** The lyophilized protein can be stored at −20°C up to 3 months.

### Heparin-induced aggregation of Tau2N4R

**Timing: 3 days**

This section describes a detailed protocol for *in vitro* heparin-induced aggregation of Tau2N4R protein.

57. Dissolve the lyophilized Tau2N4R in 25 mM HEPES buffer, pH 7.4. Measure the protein concentration based on absorbance at 280 nm.

***Note:*** Extinction coefficient for Tau2N4R is 7450 M^−1^cm^−1^.

58. Charge a 0.65 mL Eppendorf tube with 35 μL of aggregation assay at the final concentration: 20 μM Tau2N4R, 5 μM heparin, 20 μM ThT, 5 mM DTT, 50 mM NaCl, 25 mM HEPES, pH 7.4.

***Note:*** Use stock solutions of 1 M NaCl, 100 mM DTT, 110 μM heparin, 150–300 μM ThT. All stock solutions are in 25 mM HEPES, pH 7.4.

***Note:*** Use NaCl to minimize the non-specific binding of Tau2N4R protein, use DTT to keep the reaction at a reductive environment, use ThT as a signal for aggregation and use heparin to induce the aggregation.

***Note:*** Add components in the following order for better reproducibility in fibrillation. DTT, NaCl and ThT in HEPES buffer are added first, followed by protein solution, and heparin added last to induce the aggregation.

**CRITICAL:** All solutions are freshly prepared and filtered through a 0.22 μm syringe filter prior to use.

**Figure 4:**
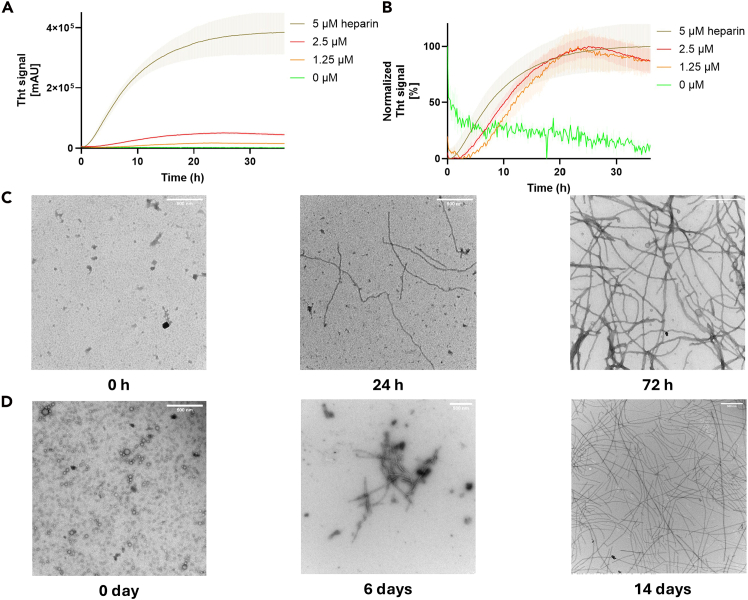
Co-factor induced or cofactor-free aggregation of Tau2N4R (A) ThT assay of heparin-induced aggregation of Tau2N4R at various concentrations of heparin. (B) Normalized ThT assay of heparin-induced aggregation of Tau2N4R at various concentrations of heparin, n = 3. The error bars represent SEM. (C) Negative staining TEM images of heparin-induced Tau2N4R fibrils (50 μM monomers) from the saturated ThT-monitored reactions. The scale bars correspond to 500 nm. (D) Negative staining TEM images of cofactor-free Tau2N4R fibrils (6 mg/mL monomers) in 10 mM sodium phosphate, pH 7.4, 10 mM DTT, and 20 mM MgCl_2_. The scale bars correspond to 500 nm.
61. Perform negative staining TEM to visualize fibrils after 3 days, Figure 4C.

***Note:*** If the assay is not working, see troubleshooting, problem 4.

59. Introduce 10 μL of the reaction to each well of a 384-well plate (Greiner BioOne, PS, F Bottom, small volume, HiBase, ref. 784900).
  a. Seal the plate with polyester adhesive film (VWR, Cat. No. 89134-430).
  b. Induce aggregation at 37°C with only shaking for 30 s before the first reading using the ‘low’ setting of shaking speed in SpectraMaxiD5 (Molecular Devices).
60. Collect the ThT signal using wavelength: Ex 440 nm, Em 480 nm, every 10 min over 72 h. Average the ThT signal from three wells, Figures 4A and 4B.

### Cofactor-free aggregation of Tau2N4R

**Timing: 12–15 days**

This section describes a detailed protocol for *in vitro* cofactor-free aggregation of Tau2N4R protein.

62. Dissolve the lyophilized Tau2N4R in 10 mM sodium phosphate, pH 7.4, and 10 mM DTT.
  a. Concentrate the sample using a 3 kDa concentrator.
  b. Achieve a concentration of ∼15 mg/mL, based on absorbance at 280 nm.

***Note:*** Centrifuge the protein at 10,000 × *g* for 10 min to remove any aggregates formed, then recheck the absorbance of the supernatant at 280 nm to determine the protein concentration.

63. Add the other components of the aggregation buffer to the protein to maintain the final concentration mentioned for each component. Final protein concentration should be 6 mg/mL.

***Note:*** Use stock solutions of 1 M MgCl_2_, 1 M DTT and 100 mM sodium phosphate, pH 7.4, to supplement sodium phosphate contributed from the 10 mM sodium phosphate, pH 7.4, 10 mM DTT buffer in protein stock to achieve a total concentration of 10 mM sodium phosphate.

***Note:*** Follow the order of adding components to the protein for better reproducibility in fibrillation. Add DTT, sodium phosphate, and water to the protein, respectively. Finally, add MgCl_2_ slowly to avoid precipitation.

**CRITICAL:** Solutions are made fresh and filtered through a 0.22 μm syringe filter prior to the experiment.

64. Introduce 50 μL of the reaction to each well of a 384-well plate having 6 nanobeads/well (800 μm Silica Beads, OPS Diagnostics).
  a. Seal the plate with polyester adhesive film (VWR, Cat. No. 89134-430).
  b. Induce aggregation at 37°C with orbital shaking of 200 rpm (1 min shaking on, 1 min shaking off) in VANTAstar Flexible Multi-mode Microplate Reader (BMG Labtech).
65. Perform negative staining TEM to visualize fibrils after 12–15 days.

***Note:*** If the assay is not working, see troubleshooting, problem 4.

***Note:*** Due to the long fibrillation time, ThT kinetics of aggregation may not be continuously monitored.

### Sample preparation for negative staining transmission electron microscopy imaging

**Timing: 3–5 h**

This section describes a detailed protocol for sample preparation of Tau fibrils for TEM imaging.

66. Glow discharge grids (Formvar/Carbon 200 Mesh, Copper Electron Microscopy Sciences) with the carbon face up (45 s, 50 mA).

***Note:*** Glow-discharged grid should be used within 2 h for sample application.

67. Apply 5 μL of sample (undiluted for heparin-induced Tau2N4R fibrils and 10 × diluted in water for cofactor-free Tau2N4R fibrils) to the carbon face of the grids.
68. Incubate the samples for 1 min on the grids.
69. Side-blot using a Whatman filter paper to remove extra samples.

***Note:*** Grids should not be completely dried in this step.

70. Apply ∼100 μL of filtered water to the carbon face of the grid and side blot as before.
71. Apply 3.5 μL of 2% uranyl acetate solution to the carbon face of the grids, stain the sample for 1 min and then remove the extra stain solution completely by side-blotting.
72. Air-dry the grids for 5 min, and the grids are now ready for imaging on the microscope.
73. Acquire TEM images in high-end TEM microscopes such as FEI Tecnai T12 Spirit Transmission Electron Microscope, Figures 4C and 4D.

***Note:*** If the TEM images are poor-quality due to fibril clumping, see troubleshooting, problem 5.

## Expected outcomes

Typically, this protocol yields approximately 8–12 mg of Tau2N4R protein after all the purification steps out of 10–12 g of cell pellet obtained from 4 liters of bacterial culture. This strategy provides an optimized framework for obtaining an ultrapure Tau2N4R, as demonstrated by mass spectroscopic analysis in Figure 3, thereby minimizing stochasticity in fibrillation reactions. Moreover, this protocol ensures a high yield of the protein, which is sufficient to set up cofactor-free fibrillation that requires milligram-scale Tau monomers. These high-concentration co-factor-free fibril preparations set the stage for high-resolution structural studies, anticipating Alzheimer’s disease-relevant folds of *in vitro* fibrils in the presence of appropriate cofactors or conditions.

## Limitations

This protocol has been successfully applied to the purification of large-scale bacterial cultures, routinely yielding milligram quantities of high-quality full-length Tau2N4R. The resulting protein preparations are devoid of detectable impurities, including cleaved His-SUMO tag, His-SUMO-Tau2N4R, and residual His-Ulp1 protease, as assessed by SDS-PAGE and mass spectrometry. The high degree of purity and molecular homogeneity achieved using this workflow enables its direct use in downstream biophysical and aggregation assays without the need for additional steps.

Using Tau2N4R prepared by this protocol, we reproducibly initiated *in vitro* fibrillation reactions across multiple independent protein preparations. Nonetheless, as has been widely observed for Tau aggregation assays, fibrillation displays an inherent degree of stochasticity, reflected in variability in lag times, elongation kinetics, and final fibril yield. This variability is particularly pronounced under cofactor-free conditions, where fibril nucleation and growth are highly sensitive to subtle differences in solution conditions, handling, and incubation history. Moreover, Tau2N4R from our preparation through *E. coli* expression is devoid of any post-translational modifications, unlike the proteins from the SF9-insect protein expression system, which can have impact in its fibrillation kinetics and morphology.

For cofactor-free fibrillation, we followed a previously reported protocol developed by Scheres and co-workers for several truncated tau constructs, with minor modifications tailored to full-length Tau2N4R.^11^ This approach was chosen with the expectation of promoting fibril morphologies relevant to Alzheimer’s disease. However, under the conditions tested, this protocol did not yield Alzheimer-like helical fibrils from Tau2N4R, as assessed by negative-stain transmission electron microscopy. Instead, the resulting assemblies largely lacked the characteristic twisted or paired helical morphology associated with Alzheimer’s disease tau filaments and predominantly yielded straight filaments.

These observations underscore both the robustness of the purification protocol and the intrinsic sensitivity of tau fibrillation outcomes to construct length, sequence context, and aggregation conditions. In particular, these observations highlight the challenges of recapitulating disease relevant fibril polymorphs from full-length tau in the absence of cofactors, post-translational modifications, or cellular components.

## Troubleshooting

### Problem 1

Low plasmid transformation efficiency in competent cells (see step 5 and 30).

### Potential solution

• The quality of competent cells can vary from batch to batch and can impact transformation efficiency. Optimizing the plasmid amount as protocol suggested to avoid cellular toxicity.
• Thaw the competent cells on ice.
• During the transformation, avoid rigorous pipetting or vortexing which cause competent cell death.

### Problem 2

In the His-SUMO tag cleavage step, the reaction is not complete after adding Ulp1 protease and the following purification step is affected. This is mostly because of the low activity or inactivity of Ulp1 protease (see step 49).

### Potential solution

• Maintain a 4°C temperature throughout the Ni-IMAC purification step of Ulp1 protease.
• Store the protein at −80°C for long-term storage (not at −20°C) in 100–200 μL aliquots to avoid repeated freeze-thaw cycles. Thaw the aliquots on ice bath before use.

### Problem 3

The His-SUMO tag cleavage is turbid, and it is causing issue to the HPLC purification (see step 50).

### Potential solution

• Centrifuge the tube at 23°C, 4,000 × *g* for 10 min and take the supernatant to the following steps.
• Add 5 mL of 6 M Guanidine to the crude reaction to make it clear right before HPLC.

### Problem 4

The heparin-induced ThT assay or cofactor-free aggregation assay is not working or is not reproducible (see step 61 and 65).

### Potential solution

• Make sure all the solutions including heparin, DTT, ThT, MgCl_2_ and buffer are freshly prepared and are filtered through a 0.22 μm filter prior to use.
• Different batches of heparin can perform differently in the heparin-induced aggregation. The same heparin should be used to maintain the reproducibility of the assay.
• The protein and ThT concentration should be tested each time before the assay using the Nanodrop.

### Problem 5

Fibril clumping leads to poor-quality TEM images (see step 73).

### Potential solution

• Sonicate the fibrils for 2 min on ice to minimize the heating and disperse the fibrils before applying to the grids.
• Increase the sonication time to 10 min if the fibrils are stored for a long time before applying them to the grids.
• Store the fibrils at 23°C or 4°C for long-term storage. Freezing the fibrils might cause irreversible clumping.

## Resource availability

### Lead contact

Further information and requests for resources and reagents should be directed to and will be fulfilled by the lead contact, Maciej A. Walczak (maciej.walczak@colorado.edu).

### Technical contact

Technical questions on executing this protocol should be directed to and will be answered by the technical contacts, Ruiheng Jing (ruiheng.jing@colorado.edu) and Sayanta Mahapatra (sayanta.mahapatra@colorado.edu).

### Materials availability

All materials generated in this study, including expression plasmids for His-SUMO-Tau2N4R and purified Tau2N4R protein, are available from the corresponding author upon reasonable request. Distribution of materials may require completion of a material transfer agreement (MTA) and is subject to institutional policies.

### Data and code availability

This protocol does not include the generation or analysis of large datasets or custom code. All data relevant to the validation and application of the protocol are included in the published article and its supplementary information. No new code was generated for this study.

## Acknowledgments

This work was supported by the 10.13039/100000049National Institute on Aging (RF1AG079294 and R01AG087295). We thank the CU-Boulder Electron Microscopy Facility and Garry Morgan for assistance with TEM experiments. We thank Dr. Taylor Minckley (University of Colorado) for assistance with the plasmid scheme on panel A.

## Author contributions

R.J., S.M., and M.A.W. designed the experiments. R.J. performed HPLC purification, SUMO cleavage, and ThT fibrillization assays. S.M. and A.B. carried out protein expression and purification. S.M. and M.H. performed transmission electron microscopy imaging. M.A.W. directed the research, secured funding, and provided administrative support. All authors contributed to writing and revising the manuscript.

## Declaration of interests

The authors declare no competing interests.

## Supplemental information

Document S1. Extended Figure S1 Representative HPLC and MS data from multiple tau2N4R purifications (A) Normalized ESI(+) mass spectra (300–2000 m/z) of HPLC-purified Tau2N4R protein from the second repeat. (B) Normalized total ion chromatography of SUMO tag cleavage reaction from the second repeat. (C) Normalized ESI(+) mass spectra (300–2000 m/z) of HPLC-purified Tau2N4R protein from the third repeat. (D) Normalized total ion chromatography of SUMO tag cleavage reaction from the third repeat.
