# Supplementary figures and images for: Protocol for expressing and purifying recombinant full-length Tau by combining affinity chromatography with preparative HPLC
Source: STAR Protoc. 2026 Jun 16;7(3):104617. doi: 10.1016/j.xpro.2026.104617 (PMC13292553; doi:10.1016/j.xpro.2026.104617)

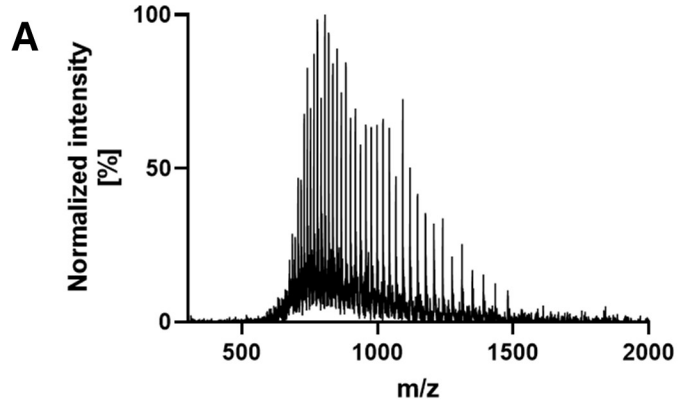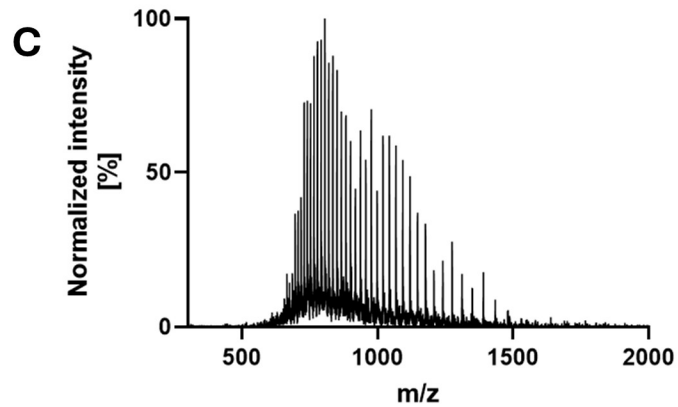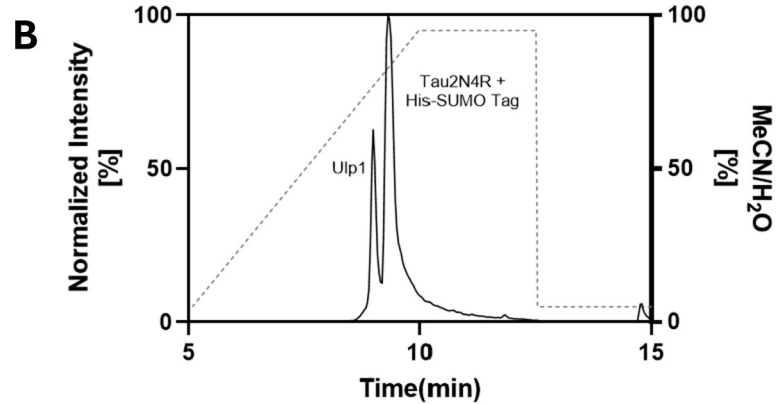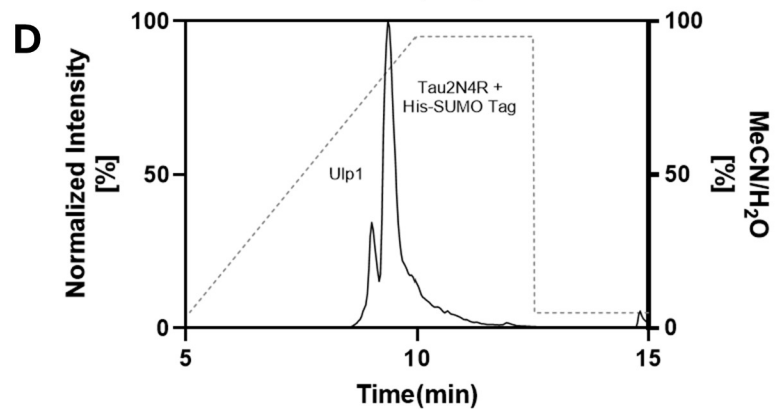

Supplement: Document S1. Extended Figure S1 Representative HPLC and MS data from multiple tau2N4R purifications — (A) Normalized ESI(+) mass spectra (300–2000 m/z) of HPLC-purified Tau2N4R protein from the second repeat. (B) Normalized total ion chromatography of SUMO tag cleavage reaction from the second repeat. (C) Normalized ESI(+) mass spectra (300–2000 m/z) of HPLC-purified Tau2N4R protein from the third repeat. (D) Normalized total ion chromatography of SUMO tag cleavage reaction from the third repeat. [file mmc1.pdf]
